# Psychological factors associated with inflammatory bowel disease

**DOI:** 10.1093/bmb/ldab010

**Published:** 2021-05-31

**Authors:** M P Eugenicos, N B Ferreira

**Affiliations:** Department of Gastroenterology, Western General Hospital, The Univesity of Edinburgh, Crewe Road South, Edinburgh EH4 2XU, UK; Department of Social Sciences, Clinical and Health Psychology, University of Nicosia, P.O. Box 24005, CY-1700, Cyprus

**Keywords:** inflammatory bowel disease, Crohn’s disease, ulcerative colitis, anxiety, depression

## Abstract

**Background:**

Both depression and anxiety are identified as significant experiences in inflammatory bowel disease (IBD); whether these are a consequence of the disease or an active contributor to the disease remains controversial. This review aimed to identify and critique recent evidence regarding mental health in IBD.

**Sources of data:**

Pubmed^Ⓡ^, Ovid^Ⓡ^, Embase^Ⓡ^, EBSCO PsychInfo and Google-Scholar were searched within the last 5 years (2016–2020).

**Areas of agreement:**

Overall, both depression and anxiety affect disease activity, relapse and healthcare utilization.

**Areas of controversy:**

There is some controversy on whether depression and anxiety affect IBD outcomes differently depending on IBD subtype.

**Growing points:**

The data support the need for depression and anxiety assessment to be incorporated in the routine management of IBD patients; prompt psychiatric and psychological management may ultimately reduce disease activity, relapses and healthcare costs.

**Areas timely for developing research:**

More longitudinal research may further enlighten the role of depression and anxiety in IBD. Similarly, randomized controlled trials to investigate and clarify the effect of psychiatric/psychological management on IBD outcomes.

## Introduction

Inflammatory bowel disease (IBD), comprises two primary forms: Crohn’s disease (CD) and ulcerative colitis (UC). IBD is characterized by an unpredictable, chronic, relapsing and remitting inflammation course of the gastrointestinal tract.^1^^,^^2^ IBD has emerged as a global disease; epidemiological studies indicate that there may be 3.2 million affected individuals in Europe, over 2 million in North America and millions more worldwide. This number has been noted to be increasing in the newly industrialized countries as a result of the westernization of lifestyle.^3^^,^^4^ The prevalence of IBD is expected to further increase due to the early age of diagnosis.^3^ The health economic burden with IBD is very high, with direct healthcare costs of billions/year in Europe.^4^^,^^5^

The impact of IBD on patients’ quality of life is particularly significant and well documented.^6^ The effects of IBD on a patients’ life may be extensively detrimental due to the variety of severe symptomatology (i.e. frequent, bloody diarrhoea, with the urgency of defaecation and faecal incontinence, fatigue, abdominal pain and weight loss), the early onset of the disease (between the ages of 15 and 30), the fluctuating feature of the disease course and the lack of a cure.^7^ IBD is considered to present a negative impact on patients’ ability to perform daily routines which may lead to frequent sick-leaves, and unemployment.^3^^,^^4^ Indeed, IBD presents a significant impact on one’s mental health. Feelings of shame, isolation and body dissatisfaction, which compromise psychosocial functioning, are often reported as being increased by the experience of having the disease.^8^^,^^9^ As a result of these experiences, IBD patients are seen as being more susceptible to develop depression or anxiety. Recently reviewed studies^10^ have shown a significant co-morbidity of IBD with depression and/or anxiety disorders (affecting one in five patients) when compared with healthy controls. However, the same study poses the controversial question of whether anxiety and depression lead to or are merely a consequence of having IBD.

Prior studies suggested that psychiatric disorders can be seen as predictors of active disease, relapses and healthcare usage rather than a consequence.^11^ Depression and anxiety could be seen as part of a self-perpetuating cycle of inflammation and mental health comorbidities that keeps the disease activity constantly fluctuating.^12^

In light of this hypothesis, several psychiatric and psychological interventions have been developed to try and target depression and anxiety as a way to break this cycle and improve IBD outcomes. However, recent reviews presented mixed success on the measured outcome for both psychiatric^13^ and psychological.^14^ Nevertheless, there is a growing consensus that depression and anxiety should be addressed and managed in the context of IBD.^10^^,^^15^

Depressive symptomatology in IBD may influence disease activity and course.^11^ Systematic reviews highlighted the impact of mental comorbidities on the disease-related outcomes.^10^ In recent years, there have been multiple advances with a rapid expansion in the armamentarium of therapies available to treat IBD, with new biologic and small molecule therapies.^16^^,^^17^ Moreover, additional protocols are being presented to optimize the application and optimal usage of these medications with a view to reducing hospitalization and surgery rates over time.^2^ Yet, despite previous recommendations, there is a lack of a systemic approach to mental health screening and treatment. The role of psychotherapeutic interventions in IBD remains controversial.^14^ Thus, the aim of this review was to identify recent studies that emerged in the ‘new biological era’, highlight and critique the current approach to managing IBD in 2020.

Given that, a systematic review in 2016,^10^ reported a lack of evidence to conclude whether depression and anxiety are predictors of IBD outcomes this review endeavours to respond to the following controversial questions:

1. Review any new evidence since 2016 that addresses whether anxiety and depression are predictors of IBD activity;
2. Examine the potential effect of depression and anxiety on key IBD outcomes of:

- disease activity,
- relapse/recurrence and
- healthcare usage

## Materials and methods

This systematic review was conducted following the guidelines adhered to preferred reporting items for systematic review and meta-analysis—PRISMA (www.prisma-statement.org). Thus, the review followed a standardized reporting system. Previous systematic reviews searched data published up to 2015, therefore the search strategy applied addressed the last 5 years. The methods including assessment of bias risk and justification of study heterogeneity were decided before the review was started.

The literature search was conducted in June and July 2020 by the first author and reviewed and agreed by the second author; the following inclusion and exclusion criteria were applied:

*Inclusion criteria*

1. IBD studies, including CD and/or UC and/or indeterminate colitis—diagnosis based on well-established criteria;
2. IBD studies included only adult population;
3. IBD studies published in peer-reviewed journals between 2016 and 2020;
4. IBD studies that examined psychological factors that included anxiety, depression and/or stress, and the main outcomes of disease activity, relapse/recurrence or healthcare use;
5. IBD studies using both prospective and retrospective methodologies, where the relation between the main variables were examined.

*Exclusion criteria*

1. Studies that did not refer to psychological factors; either anxiety or depression;
2. Studies published in languages other than English;
3. Studies published before 2016; looking at the most recent evidence;
4. Conference abstracts;
5. Articles with incomplete data or insufficient protocol presented;
6. Case reports/studies;
7. Animal studies;
8. Paediatric/adolescent studies;
9. Reviews or Editorial articles;
10. Studies with inadequate measurement of any of the variables of interest.

### Search methodology

A comprehensive search of the literature was conducted. Relevant studies were enlisted via electronic databases including Pubmed^Ⓡ^, Ovid^Ⓡ^, Embase^Ⓡ^, EBSCO PsychInfo and Google-Scholar. Additional papers were identified by searching citations of the included papers. The search strategy used terms such as ‘IBD OR Inflammation AND Bowel; Psychol^*^ AND Interventions AND factors AND IBD; Psych^*^ comorbidity AND IBD; Mental health AND IBD’. Only articles in English were reviewed, due to the inability to have them translated from other languages. The articles were screened by both authors who agreed on the inclusion and exclusion studies and criteria.

### Data collection and analysis

The systematic review was based on the PRISMA collection guidelines. Once the data were collected the articles were screened to verify whether the studies included and/or addressed any of the controversies/questions mentioned in the Introduction section.

The studies were predicted to have large heterogeneity, due to different group participants, methodologies, outcome measures and settings. Therefore, no meta-analysis was performed. The selection of the studies followed standardized recommendations of critical appraisal and included the appropriate selection of participants, control and measurement of variables.

### Data extraction and synthesis

The data extraction was performed by the second author and was reviewed for accuracy by the first author. The data extraction used a pre-formatted table to collect information on authors, year of publication, country of origin, design, sample size, participant characteristics and outcome measures. Due to the diversity of the study designs and measures used, the findings were presented in a narrative synthesis.

## Results

**Fig. 1:**
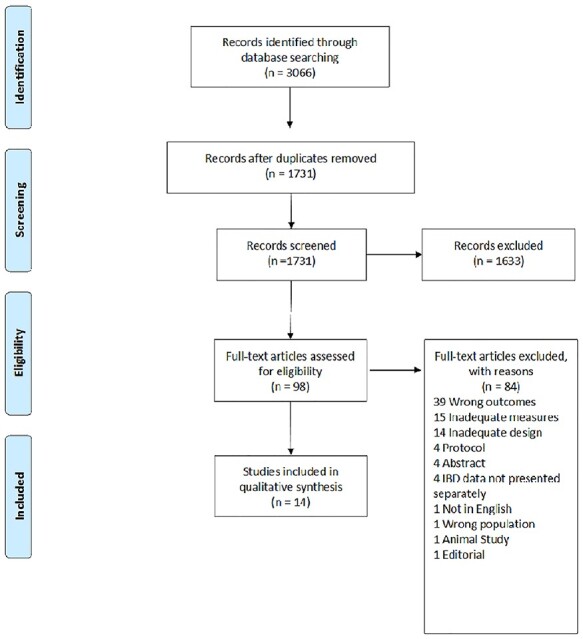
PRISMA chart.

Database searches yielded an initial pool of 3066 articles which were reduced to 1731 once duplicates were removed. Title and abstract screening excluded a further 1633 articles leaving 98 articles for full-text screening. Of these, 14 studies were selected in the qualitative data synthesis and are presented in the PRISMA flow chart (Fig. 1).

### Article characteristics

The studies presented evidence from sample sizes ranging from 120 to 4314 patients with a total collective sample of 14 111 (7829 CD, 6226 UC, 56 IBD-Undetermined). There was a variety of designs reported, with five studies using either a prospective either cross-sectional or longitudinal design and four studies using a retrospective cohort design. Most studies were conducted either in the UK (*n* = 4) or the USA (*n* = 4), with two studies from China and one study each from Italy, Germany, Brazil and Australia. As regards the outcomes of interest, nine articles addressed disease activity, three addressed healthcare usage and two addressed relapse/recurrence.

### Key findings/summary of evidence

The main features of the selected articles are summarized in Table 1. This shows that depression, anxiety and the outcomes of interest were markedly depended on the study methodology. Retrospective studies were more likely to report associations between the outcomes of interest with depression and/or anxiety, whereas the prospective studies were inconclusive. Some prospective studies reported association between depression and/or anxiety with relapse and healthcare utilisation.

In this section, we will summarize the data according to outcomes for ease of interpretation.

### Depression and anxiety as predictors of disease activity

Cross-sectional data from five studies present a mixed picture. Two studies^17^^,^^18^ suggest that both depression and anxiety contribute to an increase in disease activity in both CD and UC. However, two further studies^19^^,^^20^ rule out the role of anxiety, suggesting that only depression has a predictive role in disease activity. More controversially, the same two studies have different findings when comparing populations; one suggesting that depression influences disease activity in CD,^19^ whereas the other suggest that depression influences disease activity in UC.^20^ A further study^21^ supports the role of depression as a predictor of disease activity in CD; a limitation of this study is that it measured only depression in CD. However, its added strength is in showing the impact of depression beyond traditional measures of disease activity, but also on endoscopic and histological indicators. Conversely, it is important to emphasize that this is a cross-sectional study, so causation cannot be inferred. A study^22^ using a retrospective cohort analysis reports that depression influences both CD and UC disease activity. However, the sample was relatively small (*n* = 348) and limited in that it addressed only depression.

The studies with longitudinal prospective designs report quite disparate findings. One study^23^ shows no real influence of depression or anxiety in disease activity or on markers-of-development of disease activity (e.g. increased steroid prescriptions). Another study by the same group^24^ shows that only anxiety influences disease activity but not depression. Moreover, this study suggests that the relation between anxiety and disease activity might be mutually influenced by the self-perpetuating cycle of inflammation and mental health model.^12^ Another longitudinal study^25^ in CD patients confirms that depression influences disease activity and the number of hospitalizations. Overall the studies presented in this section, emphasize the importance of looking at CD and UC populations separately.

**Table 1:** Selected studies characteristics & findings

**Author, year & country**	**Study design**	**Sample**	**Measures**	**Main findings**	**Comments**
**Outcome variable: disease activity**					
Calixto *et al.*, 2018 Brazil	Cross-sectional Retrospective	IBD patients:120 CD:69 UC:51 Female:70 Mean age:41 yrs	Anxiety and depression: HADS^^*^^ CD activity: HBI^^*^^*^^ UC activity: Truelove Index	Disease activity in CD correlated with depression (*P* < 0.001) but not anxiety (*P* = 0.276) No association between UC severity and Depression (*P* = 0.127), or Anxiety (*P* = 0.661)	Depression seems to only play a role in CD disease activity.
Fu *et al.*, 2020 China	Cross-sectional Retrospective	IBD patients:199 CD:113 UC:86 Female:85 Mean age:35 yrs	Anxiety and depression: HADS^^*^^ CD activity: HBI^^*^^*^^ UC activity: SCCAI^^*^^*^^*^^	Positive correlation between Disease activity (*P* < 0.001) with both Anxiety (*r* = 0.544) and depression (*r* = 0.595)	Both depression and anxiety seem to have a role in disease activity
Geiss *et al.*, 2018 Germany	Retrospective cohort analysis	IBD patients:348 CD:228 Females: 120 Median age: 38 UC:120 Females: 61 Median age: 41	Depression: PHQ-9^^*^^*^^*^^*^^ CD activity: HBI^^*^^*^^ UC activity: SCCAI^^*^^*^^*^^	Positive correlations (*P* < 0.001) Depression with disease activity in CD (*r* = 0.621) and UC (*r* = 0.386)	Depression seems to play a role in disease activity
Gracie *et al.*, 2018a UK	Longitudinal-prospective Follow-up: 2 year	IBD patients:360 CD:200 UC:160	Anxiety and depression: HADS^^*^^ CD activity: HBI^^*^^*^^ UC activity: SCCAI^^*^^*^^*^^ Objective markers of activity: 1. steroid therapy/flare up 2. escalation of therapy 3. hospitalization 4. intestinal resection	Anxiety and depression did not predict any of the objective markers of IBD activity	Objective markers of IBD activity were not influenced by either anxiety or depression.
Gracie *et al.* 2016 UK	Cross-sectional Retrospective	IBD patients:356 CD:191 Females: 119 Mean age: 47 UC:165 Females: 89 Median age: 51	Anxiety and depression: HADS^^*^^ CD activity: HBI^^*^^*^^ UC activity: SCCAI^^*^^*^^*^^	Depression was associated with increased UC activity (OR 1.21 per 1- point increase HADS; 95% CI 1.02–1.44) No correlation between depression and CD activity; No correlation between anxiety and UC or CD activity (95% CI contained 0)	Depression seem to play a role in UC activity but not CD activity.
Gracie *et al.*, 2018b UK	Longitudinal Prospective Follow-up: 2 year	IBD patients:405 CD:239 UC:166	Anxiety and depression: HADS^^*^^ CD activity: HBI^^*^^*^^ UC activity: SCCAI^^*^^*^^*^^ Objective markers of activity: 1. steroid therapy/flare up 2. escalation of therapy 3. hospitalization 4. intestinal resection	Baseline disease activity predicted development of abnormal anxiety (HR 5.77, 95% CI 1.89–17.70) but not depression Baseline abnormal anxiety predicted: a). the need for steroid therapy (HR 2.08, 95% CI 1.31–3.30), b). escalation of medical therapy (HR 1.82, 95% CI 1.19–2.80), but not hospitalization, or bowel resection, or disease activity (all 95% CI contained 0) Baseline abnormal depression did not predict any of the disease-related variables (all 95% CI contained 0)	The study suggests a bi-directional influence of anxiety on disease activity but not depression.
Leone *et al.*, 2019 Italy	Cross-sectional Retrospective	IBD patients:201 CD:95 UC:106 Females: 99 Mean age: 41	Anxiety and depression: SCL-90-R^†^ CD activity: HBI^^*^^*^^ UC activity: Mayo score	Patients with active disease had higher depression (*P* = 0.001) and anxiety (*P* = 0.013) levels as compared with patients in remission	Both anxiety and depression seem to be associated with disease activity
Gaines *et al.*, 2016 USA	Longitudinal Prospective Follow-up: 1 year	CD:2144 Females: 120 Median age:43	Depression: PROMIS^††^ CD activity: SCDAI^†††^	Significant associations between baseline depression and follow-up: Active CD (*P* = 0.001, *df* = 2) Hospitalizations (*P* = 0.03, *df* = 2) No significant associations between baseline depression and follow-up: Abdominal surgeries (*P* = 0.34, *df* = 2) Anti-TNF prescription (*P* = 0.15, *df* = 2)	Baseline depression seems to influence disease activity and hospitalizations at 12 month follow-up.
Tang *et al.*, 2019 China	Cross-sectional Retrospective	CD:133 Females: 39 Median age:34	Depression: HADS^^*^^ CD activity: HBI^^*^^*^^ SES-CD ^††††^ GHAS^‡^	Depression correlated with: HBI (*r* = 0.250, *P* = 0.004) SES-CD (*r* = 0.220, *P* = 0.011) GHAS (*r* = 0.181, *P* = 0.037)	Study shows increased relative clinical activity, endoscopic scoring, and histological grading in depressed CD patients
**Outcome measure: relapse**					
Kochar *et al.* 2018 USA	Retrospective database analysis Follow-up CD: 22 months UC: 24 months	IBD patients: 4314 CD:2798 Females: 1567 Mean age: 41 UC:1516 Females: 743 Median age: 42	Depression: PHQ-8^^*^^*^^*^^*^^ CD activity: HBI^^*^^*^^ UC activity: SCCAI^^*^^*^^*^^	CD patients with baseline depression were at an increased risk: for relapse (RR: 2.3; 95% CI: 1.9–2.8), surgery, or hospitalization (RR: 1.3 95% CI: 1.1–1.6) at follow-up. UC patients with baseline depression were also at increased risk: for relapse (RR: 1.3; 95% CI: 0.9–1.7), surgery, or hospitalization (RR: 1.3; 95% CI: 1.1–1.5) at follow-up	Baseline depression is associated with a higher risk of relapse
Mikocka-Walus *et al.*, 2016	Longitudinal prospective cohort study	IBD patients:2007 CD:1122 Females: 610 Median age 42 UC: 885 Females: 427 Median age 41	Anxiety and depression: HADS^^*^^ CD activity: CDAI^‡‡^ UC activity: MTWAI^‡‡‡^	Depression correlated with shorter time to relapse in CD (*P* = 0.007) & UC (*P* = 0.005) Total sample (*P* = 0.0000001) Anxiety correlated with shorter time to relapse in CD (*P* = 0.031) but not UC (*P* = 0.066) Total sample (*P* = 0.0014)	Depression and anxiety seem to be associated with clinical recurrence in IBD
**Outcome measure: healthcare utilization**					
Lores *et al.*, 2020 Australia	Longitudinal prospective cohort study Follow-up: 12 months	IBD patients:335	Anxiety and depression: HADS^^*^^ Psychological distress: Kessler 6 scale Healthcare utilization: Emergency department attendance Hospital admissions	Increased attendance to emergency department was predicted by: Higher depression (*P* = 0.02) (OR: 1.08, 95% CI: 1.01–1.14) Higher anxiety (*P* = 0.02) (OR: 1.07, 95% CI: 1.01–1.13) Psychological distress (*P* < 0.01) (OR: 1.06, 95% CI: 1.02–1.11, Hospital admissions were predicted by: Higher depression (*P* = 0.03) (OR: 1.07, 95% CI: 1.01–1.13, Psychological distress (*P* = 0.02) (OR: 1.05, 95% CI: 1.01–1.10, But not anxiety (*P* = 0.15)	Mental health issues were associated with higher healthcare costs
Navabi *et al.* 2018 USA	Retrospective cohort study Follow-up: 12 months	IBD patients:432 CD:256 UC:132 Indeterm.colitis: 44 Females: 228 Mean age: 42	Anxiety and Depression: HADS^^*^^ Healthcare utilization: Emergency attendance Hospital admissions Imaging studies Surgeries	Patients with raised anxiety and/or depression scores were more likely to: Present to emergency room (*P* < 0.05) Be hospitalized (*P* < 0.05) Have more imaging tests (*P* < 0.05) But not more surgeries (*P* = 0.06)	Both anxiety and depression seem to predict healthcare usage.
Poojary *et al.*, 2017 USA	Retrospective cohort study Follow-up: 30 days	UC:2757	Depression measure: Healthcare Research and Quality-Web ICD-9-CM Healthcare utilization:: 30-day unplanned readmission	Depression was significantly (OR1.40, 95% CI, 1.16–1.66) associated with unplanned readmission	Depression significantly increased the odds of readmission by 40%

The associations of depression, anxiety and the outcomes of interest markedly depended on the study methodology. Retrospective studies were more likely to report associations between the outcomes of interest with depression and/or anxiety, whereas the prospective studies were inconclusive. Some prospective studies reported association between depression and/or anxiety with relapse and healthcare utilisation.

^^*^^Hospital Anxiety and Depression Scales: HADS.

^^*^^*^^Harvey Bradshaw Index: HBI.

^^*^^*^^*^^Walmsley Simple Clinical Colitis Activity Index: SCCAI.

^^*^^*^^*^^*^^Patient Health Questionnaire—8 or 9 items: PHQ-8 or PHQ-9.

^†^Symptom checklist-90-R: SCL-90-R.

^††^Patient-reported outcomes measurement information system: PROMIS.

^†††^Short Crohn’s Disease Activity Index: SCDAI.

^††††^Simple endoscopic score for Crohn’s disease: SES-CD.

^‡^Global Histological Disease Activity Score: GHAS.

^‡‡^Crohn’s Disease Activity Index: CDAI.

^‡‡‡^Modified Truelove and Witts Severity Index: MTWAI.

### Depression and anxiety as predictors of relapse/recurrence

We identified two studies addressing the impact of depression and/or anxiety on relapse. A study with a retrospective cohort design^26^ shows that baseline depression is associated with a higher risk of relapse at 22 and 24 months follow-up for CD and UC, respectively. Another study, using a longitudinal prospective cohort design,^27^ shows that baseline depression and anxiety are associated with a shorter time to relapse across IBD conditions. However, on separately analysing CD and UC, anxiety was not confirmed as a predictor of relapse in UC. Yet again, there seems to be a trend towards disparity of depression and anxiety influence depending on IBD subtype.

### Depression and anxiety as predictors of healthcare utilization

In total, three studies investigated the influence of depression and anxiety in healthcare utilization of IBD patients. A study using longitudinal prospective design^28^ reports that depression and psychological distress predicted a higher number of emergency presentations and admissions over a 12-month period, whereas anxiety predicted only a high number of emergency presentations. In a relatively small (*n* = 432) retrospective cohort study,^29^ patients with raised depression and anxiety levels were more likely to present to the emergency department, and/or be hospitalized, undergo more investigations, but not surgeries. A further large retrospective cohort study^30^ in UC, depression increased the odds of unplanned hospital readmission over a period of 30 days by 40%. Overall, data suggest that depression and anxiety may be associated with excess healthcare utilization.

## Discussion

This systematic review presented data on patients with mental health issues and their risk of relapse, hospitalization, and/or surgery; thus, data on increased healthcare utilization. However, controversy still exists in the way mental health influences disease activity. Most studies take an ontological stance on considering depression and anxiety as predictors of disease activity or vice-versa. The reviewed studies seem to confirm the former. Conversely, a plethora of evidence for the latter is also available in the literature.^31^ It is encouraging that at least one study^24^ took a middle ground approach and produced evidence for the mutual influence between mental health and disease activity. This is in line with the self-perpetuating model proposed elsewhere.^12^

Unanswered questions remain regarding the discrepancy of anxiety and depression role in IBD. Depression may be considered a more stable construct, perhaps more reliably measured across contexts.^32^^,^^33^ Anxiety, though, maybe more context-dependent, and widely variable across populations, depending on the measures used.^34^ The reviewed studies failed to discern if the reported anxiety is specific to IBD or whether is an epiphenomenon to other situations in the patient’s life; the hypothesis that anxiety might be related to the economic burden secondary to IBD rather than the result of the disease itself is a valid argument. This may explain the more inconsistent evidence for anxiety when compared with depression. It might be that further to general measures of anxiety such as the ones used in most of the reviewed studies like the HADS,^35^ one might want to consider more specific measures of GI anxiety such as the Visceral Sensitivity Index (VSI),^36^ which might produce a greater sensitivity to IBD specific anxiety. Further to measurement issues, the reviewed studies highlighted a potential difference in mental health issues affecting disease activity depending on the IBD subtype. Some of these differences may be specific to some individual characteristics of each IBD subtype, such as the greater risk of surgical intervention, the type of pharmacological treatment or disability caused; more studies are needed to address this differential effect.

The data reviewed were unequivocal about a strong effect of mental health on relapse/recurrence. This is likely to be associated with the known stress reactive cytokine production which is then linked to the inflammation pathway. Furthermore, there is also ample evidence^37^ that depression and anxiety are likely to affect key aspects of treatment such as medication adherence,^38^ which in turn is likely to lead to more frequent relapses. Similarly, the data reviewed all pointed to greater healthcare utilization in patients who have increased levels of anxiety and depression. It is recognized the raised levels of anxiety and depression might be connected to the aforementioned higher risk of relapse and a greater impact on disease activity; equally, this might also be attributed to poor consultation behaviour. Patients with mental health issues tend to be on the extreme ends of the spectrum; either over-consulting, using more resources,^24^ or by delaying their consultation to such extend that they are more likely to present as an emergency and be hospitalized.

There were some limitations to this review, as no meta-analysis was conducted, therefore the overall conclusions are based only on a narrative synthesis. The main reason for this, as previously noted, was that different studies considered different ontological stances regarding the cause-effect relation between IBD and mental health outcomes, thus introducing heterogeneity in their methodological approaches. Moreover, there was a lack of good quality prospective evidence, therefore any causal effects reported should be taken cautiously.

This review’s epilogue is that the role of psychiatric co-morbidities such as anxiety and depression in IBD remains controversial. Anxiety and depression rates are higher in patients with active disease as compared with those in remission.^18^ In this day and age, despite improved healthcare with novel more effective treatments^2^ higher rates of anxiety and depression influence disease activity, relapse, healthcare utilization and may be part of the main drivers in healthcare costs in IBD.^4^

## Conclusion

In this review, we aimed to provide an updated synthesis of the literature on the effect of depression and anxiety in IBD outcomes of disease activity, relapse/recurrence and healthcare utilization. On the whole, the evidence suggests that depression and anxiety influence all the aforementioned outcomes. This may warrant a systemic holistic approach to screening and treatment. The benefit of value of routine screening has been established in populations with chronic conditions and/or mental disorders.^39^ The use of Psychotherapy in IBD also remains controversial,^14^ although more recent studies showed that cognitive behavioural therapy, improves psychological distress and gastrointestinal symptoms.^40^ As evidenced by the thousands of citations initially identified for this review, the research in psychiatric comorbidities in IBD is quite extensive. However, there are ongoing problems with methodologies and study heterogeneity, thus the use of meta-analysis could not be facilitated.

Our recommendations for future research would be to include case-control, population-based studies with comparison groups for both healthy and patients with chronic illnesses; prospective, randomised controlled studies to collect data on mental health and other symptoms including pain from patients with active IBD and patients in remission. This may enlighten any cause-effect mechanism and healthcare use accordingly; it may also address the important issue of persistent pain as a feature of inactive IBD, and its relation to anxiety/depression. The use of structured clinical interviews, screening and clinical diagnostic measures that are validated will lead to more homogeneous studies. Subsequently, this will improve the quality of research in this area and accordingly enlighten medical professionals and patients as to the best approach to treatment.

## Data availability statement

No new data were generated or analysed in support of this research.

## Conflict of interest statement

The authors report no conflicts of interest.
